# Service User Experiences of How Flexible Assertive Community Treatment May Support or Inhibit Citizenship: A Qualitative Study

**DOI:** 10.3389/fpsyg.2021.727013

**Published:** 2021-09-08

**Authors:** Eva Brekke, Hanne K. Clausen, Morten Brodahl, Annika Lexén, Rene Keet, Cornelis L. Mulder, Anne S. Landheim

**Affiliations:** ^1^Norwegian National Advisory Unit on Concurrent Substance Abuse and Mental Health Disorders, Inland Hospital Trust, Brumunddal, Norway; ^2^Department of Research and Development, Division of Mental Health Services, Akershus University Hospital, Lørenskog, Norway; ^3^Department of Health Sciences, Medical Faculty, Lund University, Lund, Sweden; ^4^Flexible, Innovative Top-ambulatory Academy of Community Mental Health Service, Geestelijke Gezondheidszorg Noord-Holland-Noord, Heerhugowaard, Netherlands; ^5^Department of Psychiatry, Erasmus Medical Center (MC), University Medical Center Rotterdam, Rotterdam, Netherlands; ^6^Department of Health and Nursing Sciences, Faculty of Social and Health Sciences, Inland Norway University of Applied Sciences, Elverum, Norway

**Keywords:** flexible assertive community treatment, citizenship, integrated care, severe mental illness, substance use disorder, recovery

## Abstract

The aim of this study was to explore and describe service user experiences of how receiving services from a Flexible Assertive Community Treatment (FACT) team may support or inhibit citizenship. Within a participatory design, individual interviews with 32 service users from five Norwegian FACT teams were analyzed using thematic, cross-sectional analysis. The findings showed that FACT may support citizenship by relating to service users as whole people, facilitating empowerment and involvement, and providing practical and accessible help. Experiences of coercion, limited involvement and authoritarian aspects of the system surrounding FACT had inhibited citizenship for participants in this study.

## Introduction

Equity between groups and fair access to mental health for all are important goals from a public mental health perspective (Knifton and Quinn, 2013), recognizing health as a fundamental human right [United Nations, 2006; UN Office of the High Commissioner for Human Rights (OHCHR), 2008]. Yet, persons with severe and complex mental health care needs face challenges related to citizenship, living conditions, and fair access to health and social services (Drew et al., 2011; Norwegian Board of Health Supervision, 2019). Rights-based, holistic actions that are rooted in the experiences of people that are excluded from current mental health systems and practices, have been called for in order to address the unfair distribution of health in the population (Special Rapporteur on the right to health, 2020).

Normative notions of citizenship may expect people with disabilities to assume responsibility for becoming independent, “ideal” citizens, regardless of their actual available resources, with the risk of excluding those who do not match these expectations. On the other hand, relational, cultural and structural approaches to citizenship stress the need for building inclusive communities that support citizenship for all, while acknowledging the importance of providing acceptable living conditions in order to support genuine access to citizenship (Vandekinderen et al., 2012). Rowe's citizenship framework is rooted in sociological theory and in mental health outreach work with homeless persons in the United States (Rowe et al., 2001), partly based on the Assertive Community Treatment (ACT) model (Stein and Test, 1980). This approach concerns the participation and inclusion in society of people with mental health problems and complex needs, such as poverty, substance use, criminal justice involvement or homelessness (Ponce and Rowe, 2018). Within this framework, “citizenship” is defined as “…the person's strong connection to the 5 Rs of rights, responsibilities, roles, resources, and relationships that society offers to its members through public and social institutions and associational life, and a sense of belonging in society that is validated by one's fellow citizens” (Ponce and Rowe, 2018, p. 2). Regaining citizenship requires the person's own efforts as well as the society's ability to provide access to citizenship (Rowe et al., 2001), and a sense of belonging depends on the recognition of the individual by society. Consequently, services working to support citizenship will not only see it as their task to support the efforts of the individual and his or her immediate environment, but also to reduce barriers to citizenship in the community and society at large (Stewart et al., 2017).

Citizenship and theory originating from the recovery movement in psychiatry have many common points, such as the emphasis on civil rights and a holistic view of the person. However, while recovery theory increasingly defines recovery as a personal, social and relational process, the citizenship framework explicitly defines and emphasizes the elements that are necessary to fully participate in society (Rowe and Davidson, 2016). This resonates with approaches that see recovery as interdependent on the interpersonal relationships and social environments of the individual (Price-Robertson et al., 2017; Mudry et al., 2019). The need for relational and inclusive approaches to citizenship and recovery in mental health has been supported by studies of first-person experiences (Borg and Davidson, 2008; Vervliet et al., 2019; Brekke et al., 2020).

Norwegian health and social services have fallen short in providing integrated care for citizens with severe mental illness and complex needs such as substance use problems or homelessness. Fragmented services may be perceived as irrelevant by people with complex needs, leading to a lack of trust in the system (Landheim et al., 2017). As a response to this, Norwegian health authorities have encouraged the implementation of the Flexible Assertive Community Treatment model (FACT), which is a Dutch adaptation of ACT (Stein and Test, 1980; van Veldhuizen, 2007). The FACT model aims to provide long-term, integrated and comprehensive services to persons with severe mental illness, complex needs, and a low level of daily functioning (van Veldhuizen and Bahler, 2013). FACT is based on a bio-psycho-social approach to mental health and substance use problems, and aims to prevent admissions to inpatient treatment and promote personal recovery and participation in the community. Within a multi-disciplinary team, FACT provides evidence-based treatment of mental illness and substance use problems, practical support in everyday life, rehabilitation, and support in each person's recovery process. The model enables flexible adaptation of the intensity level of support, ranging from regular individual support to intensive support based on the person's needs.

FACT is an example of sectoral integration of services, involving a multi-disciplinary team that provides both community-based and specialized services (Goodwin, 2016). In a focus group study of experiences of service providers that collaborate with Norwegian FACT teams, participants described how FACT may form a bridge between services, providing reassurance to other actors in the service system (Trane et al., 2021). The model is also an example of people-centered integration, as it implies a focus on client know-how and community support, and close collaboration with the local community (Goodwin, 2016). Supporting participation and inclusion in the community is one of the cornerstones of the FACT model. In a recent consensus statement, the European Community-based Mental Health Service Providers (EUCOMS) Network suggested certain principles for community-based mental health care, namely human rights, public health, recovery, effectiveness of interventions, community network of care, and peer expertise (Pieters et al., 2017). The FACT model is mentioned as an example of good practice of integrated community mental health care, albeit with less conclusive evidence than the ACT model (Keet et al., 2019).

Research has provided mixed results on whether FACT may enhance social functioning (Drukker et al., 2013; Svensson et al., 2018; Kortrijk et al., 2019a) or employment rates (Kortrijk et al., 2019b). Quantitative studies have not shown convincingly that the FACT model is superior to ACT or other types of community mental health care services (Nielsen et al., 2020). The Resource Group (RG) method has been suggested as a means of improving the FACT model in order to enhance social inclusion and the involvement of patients' networks (van Veldhuizen et al., 2015). In a grounded theory study of service users in FACT with severe mental illness, emphasizing the inclusion of the RG method in FACT, the authors conclude that the combination of RG and FACT enhances recovery as a social process that involves everyday life and significant others (Tjaden et al., 2020). In a Dutch grounded theory study of 15 service users of FACT who had mild intellectual disability or borderline intellectual functioning, participants stated that being in contact with FACT had improved their daily life situation, and they valued both the relationship with staff and the practical and emotional support given by the team (Neijmeijer et al., 2020). Qualitative studies of service user perspectives in ACT have suggested that ACT may lead to reduced social isolation and solutions to daily life issues (Stuen et al., 2015) and make it more attractive to remain in treatment over time (Pettersen et al., 2014), while this has not been studied in FACT. Another study from ACT reported complex experiences including increased community integration as well as experiences of limitations and marginalization (Lofthus et al., 2018).

There is a need for knowledge about how the FACT model may support or inhibit citizenship. Exploring the perspective of service users may enhance understanding of how practitioners may work to support citizenship in useful and meaningful ways. Hence, the aim of this paper is to explore and describe service user experiences of how receiving services from a FACT team may support or inhibit citizenship.

## Materials and Methods

### Study Context

This study is part of a larger research project that investigates the implementation of the FACT model in Norway. The study context was five FACT teams in different parts of Norway with varied geographical contexts (Table 1). The five teams took part in a national evaluation of FACT that was led by the 7th author, and were deliberately selected to the research project aiming for variation in geographical context in order to reflect the large variation between rural and urban areas in Norway. The teams were positive toward participating in research, and committed to working according to the FACT manual (van Veldhuizen and Bahler, 2013). All teams had case managers (nurses and social workers), peer specialist, occupational specialist, psychologist and psychiatrist. Two of the teams (teams 4 and 5) also had a music therapist in the team.

**Table 1:** Team characteristics.

	**Population/ catchment area**	**Year established**	**Region**	**Patients**	**Team members**
Team 1	23500 / 1201 km^2^	2018	Rural Northern Norway	29	9
Team 2	18600 / 1289 km^2^	2019	Rural Northern Norway	33	10
Team 3	24000 / 4327 km^2^	2014	Rural Western Norway	40	7
Team 4	30400 / 671 km^2^	2018	Urban Eastern Norway	47	8
Team 5	58500 / 7, 5 km^2^	2013	Urban area in capital city, Eastern Norway	122	11

Norwegian mental health care has two levels of administration; municipalities run primary healthcare (municipal services), whereas hospital trusts are responsible for secondary and tertiary care (specialized services). Municipal and specialized services share responsibility for providing services to people with severe mental illness and complex needs. Community mental health care consists of community mental health centers (CMHCs), which are part of specialized services, but collaborate with municipal services. The municipalities have a large degree of autonomy, and the geographical context varies considerably, leading to variety in the organization and content of mental health and social services. There is an ongoing specialization of mental health services in Norway, which may improve the quality of services, but also increase fragmentation (Ruud and Friis, 2021).

The first Norwegian FACT team was established in 2013, implying a new way of organizing services with municipal and specialized services within one team. There are currently around 60 FACT teams in Norway. The service system surrounding FACT includes inpatient and outpatient treatment, general practitioners (GPs), welfare services, and specialized substance use services, among others (Trane et al., 2021). FACT mainly provides voluntary services, and reducing involuntary admissions is a rationale for implementing FACT and ACT (Clausen et al., 2016). However, some service users have community treatment orders (CTOs), which oblige them to comply to medication and attend appointments. The CTOs are administered by the responsible specialist (psychiatrist or clinical psychologist). In some teams, FACT team members carry out the coerced medication, while in other teams this is done by other services.

Data collection was carried out between September 2020 and February 2021, during COVID-19 restrictions. Except for shorter periods of lock-down, all FACT teams continued with outreach work. Other services, such as libraries and user-led houses, were closed for shorter periods of time.

### Design

The study has a participatory, exploratory, qualitative design. Co-production has been involved at two levels (Beresford, 2013) in order to increase the relevance and validity of the study, by including persons with varied knowledge and experiences of the phenomena being studied (Barber et al., 2011; Ness et al., 2014). At the level of service user involvement, a peer group was consulted throughout the study at regular meetings with the first and third authors. Group members are two peer support workers from different FACT teams, one person with service user experience in FACT, and one person who is active in a local peer support house. The peer group gave advice in planning the study, developing the interview guide, organizing the interviews, and understanding the results. At a participatory level, the third author, who has lived experience of receiving mental health and substance use services, has participated as a co-researcher in all stages of the study, including planning, data collection and analysis, as described in the following sections.

### Recruitment and Participants

The recruitment strategy aimed at diversity in substance use and mental health problems, experiences of coercion, age, gender, and duration of contact with services. Recruitment was organized by the peer support worker or the leader of the FACT team. Flyers with project information and contact details of the first and third authors were handed out by team members to all service users for a designated period of time. Some participants contacted us directly, some forwarded their contact details through team members, while others agreed to participate but preferred not to be in touch before the interview.

Thirty-two persons participated in the study. Participants were 21 men and 11 women between the ages of 20 and 67 years (mean age 37). Seventeen participants reported a diagnosis of psychotic disorder, eight had bipolar disorder, and seven had other mental health problems (two of whom were undergoing diagnostic assessment for psychotic disorder). Sixteen participants reported a severe substance use and/or alcohol problem at the time of the interview, and three participants were enrolled in opioid agonist therapy. Four participants reported former substance use problems that were currently under control, while 10 reported never having had problems with alcohol or substances. The length of contact with the FACT team ranged between four and 78 months (29 months on average). At the time of the interview, the intensity varied from daily to monthly contact, with most participants reporting weekly contact or more. One participant had a CTO at the time of the interview, while 25 participants had experienced compulsory admissions and/or CTOs in the past.

### Data Collection

Interviews were arranged by the peer support workers, FACT team leaders or other team members. Two interviews were canceled by the participant on the day of the interview and we ended up with 32 participants. In teams 3 and 5, interviews had to be postponed for two months due to national COVID-19 restrictions. Three participants were no longer able to participate after two months, while three others agreed to participate at this point. In teams 1, 2 and 4, interviews were conducted in person at the team's location or in the participant's home, according to each participant's preferences. In team 3, interviews were held on a secure digital platform, facilitated by a team member. In team 5, interviews were carried out by telephone because of a severe lockdown at the time, preventing team members from arranging digital interviews.

The first and third authors conducted all interviews together. Interviews were organized as semi-structured conversations based on an interview guide with open-ended questions about participants' experiences of receiving services from a FACT team. Because the first interviews mainly resulted in descriptions of positive experiences, we began to explicitly ask for negative experiences and suggestions for improving services. Interviews lasted from 28 to 79 min, and were digitally recorded and transcribed verbatim. One interview was not recorded due to participant reservations. Notes from this interview were included in the analysis.

### Analysis

Interviews were analyzed using systematic text condensation, a pragmatic method for thematic, cross-sectional analysis (Malterud, 2012). First, all transcripts were read through by the first and third author separately, looking for overarching themes. The first and third authors then met with the 7th author to discuss the themes along with text extracts to illustrate the themes. These themes and the text extracts were discussed in the advisory group, yielding a richer understanding and context of participants' descriptions. The complete transcripts were then read through systematically, and meaning units were sorted into code groups. This process was guided by the preliminary themes, but the code groups were adjusted as insight into the data increased. Each code group was then read through systematically, meaning units were arranged into subgroups within each code group, while the code groups and subgroups were adjusted throughout. Meaning units within each subgroup were reduced to an artificial quotation, maintaining the original terminology used by the participants. The code groups and condensates were discussed with the other authors in order to gain multiple perspectives. At this point, the analysis consisted of three code groups that were organized around central aspects of FACT. We have included descriptions of participants' experiences of how these aspects of FACT facilitated or inhibited citizenship. The meaning content of the condensates was then synthesized to develop an analytic text that constitutes the results section of this article, with illustrative quotations that are presented as they appear in the transcripts. Finally, the analytic text was validated by returning to the original transcripts.

### Ethical Considerations

The study was approved by the local data protection officer (ID 137850), in accordance with the Regional Committee for Medical and Health Research Ethics. Interviews were based on informed consent. Written and oral information was given to all participants before the interview started, with the opportunity to withdraw at any point. Three participants chose to have the peer support worker present during the interview, and two participants chose to have their case manager present. In these cases, the team member offered to leave the interview if the participant wanted. We always underlined that interviews were confidential, and would have no impact on their future services from FACT.

## Results

Participants described past and present experiences of barriers to citizenship, such as loneliness, poverty, and stigma. FACT may support citizenship by relating to service users as whole people, by facilitating their empowerment and involvement, and by providing practical and accessible help. Experiences of coercion, limited involvement and authoritarian aspects of the system surrounding FACT were described by the participants as inhibiting their sense of citizenship.

### Being Viewed as a Whole Person

Participants described that FACT had supported citizenship by meeting them as a whole person. Being viewed as a whole person was described as unique to FACT compared to other services, and had provided safety, insight, opportunities for recovery and participation in society as a citizen. Receiving help with different aspects of life was described as an upward spiral of hope where mental health problems became less of a restriction in everyday life, allowing participants to live less isolated and lonely lives, alienated from society. Receiving help with all one's needs from the same team was felt to be better, more secure and easier to deal with than other services they had been in contact with. One participant compared FACT to a parachute with many strong ropes, which made it safer to take a leap into challenging situations. Another participant put it this way:

“*It's great that they have this team where you have what you need. It makes things easier to deal with. (…) You feel reassured then, because it's a professional team you can relate to.”*

Several participants found it helpful that FACT advised them about participation in different activities in the community. Opportunities for enjoyable and varied everyday experiences, such as being creative, playing in a band, joining a choir, going to the gym, joining the community at a Fountain House (mental health clubhouse), and seeing other people, were described as “sifting out the painful things and filling one's cup to the brim.” Walking in the countryside was appreciated as it involved physical exercise, nature experiences, coping, being sociable in a non-demanding way, getting out of the house, seeing new places and receiving new impulses. Participating in pleasurable activities had led to better sleep, less need for medication, better mental capacity, and a sense of community belonging and normality for the participants in this study. One young participant expressed it as follows:

“*Instead of just going to the community mental health center once or twice a week, like I did before, I have someone who sees me as a whole person.”*

Another participant put it this way:

“*I've quit blaming myself so much. I've had a tendency to do self-harm. (…) Now I get a bit of exercise and I can do art and there's the social life and the professional help. It's like a really good cake recipe.”*

Living a normal and enjoyable life was described as a primary goal by several participants, but many felt labeled and left out from the community because of substance use or mental illness, particularly in small towns. It was appreciated when practitioners in FACT acknowledged and addressed experiences of labeling and exclusion. One participant had arrived for a scheduled visit at her former workplace to find that everyone had gone out for lunch. Another participant said:

“*No matter who I meet, I smile and say hi. But I'm surprised at how few people say hi back to me.”*

Being seen by FACT as a whole person had made it easier to rise above stigma, accept invitations and resume contact with old friends. Others still found it difficult to contact old friends because they did not know what to say about their life and their diagnosis. Many had lost friends to overdoses, suicide or accidents. Some participants felt lonely after quitting substances because they felt that they now had less in common with old friends who were still using drugs. Others found it unlikely that they would break with the substance use community. A young woman said:

“*I go crazy being alone, but not being alone can be too much as well. So I end up taking drugs, and the days go by, and it's an eternal system that doesn't really have any end. I don't think I'll ever get out of the drug scene. (…) But people just think about if your tests are clean. They don't give a shit what people have to say.”*

Another participant described his contact with FACT in this way:

“*They don't yell at me and tell me to take my medication or put me in belts and stuff like that. They communicate with me and give me advice, like go to the Fountain House, come and see us in FACT, have a smoke with the peer support worker, just relax and feel like a citizen, in a way. And that really means a lot!”*

Some participants expressed shame, failure and sadness related to not having a degree, family, network or job, which all increased the feeling of being alienated from society. It was appreciated when practitioners in FACT understood and addressed these phenomena. Some wanted to work, but were afraid of disappointing themselves or others if they could not do the job due to their mental health problems. Many described wanting to move on, to be seen, and to have somewhere to go to. One participant described unemployment as a state of fading away. Assistance from FACT in getting a job, adapting to the work, and moving other appointments to outside working hours, was described as valuable. One participant was critical of unpaid work training, and appreciated the focus of FACT on paid work. Another participant described the switch to working life as brutal because it implied so much contact with other people. A good relationship with one's boss was highlighted as crucial in order to remain in a job over time. One participant described the importance of work in this way:

“*I'm no good at being sociable, I'm no good at being around people. Maybe that's why I'm alone and don't have any kids. I don't know. But working with people, talking, having routines in my life, that's what I need. Getting up in the morning, working, using my body, being part of society, you know. You need to involve yourself and get a life, one that you feel you're living and want to live. And own it yourself.”*

Stable housing and a safe home had provided better quality of life, peace, and the opportunity to quit substance use. Receiving support from FACT in sorting out one's finances and getting stable housing was described as crucial. Several participants had experienced homelessness, either before or at the time of the interview, which was associated with instability, chaos and difficulty in keeping focused. A vicious circle between substance use and poor housing was described, where poor housing led to substance use and vice versa.

Family involvement in FACT was appreciated by some participants, leading to less concern among family members, improved relationships with family, and relief from difficult family relationships. Others did not want their family to be involved. Several participants described a difficult relationship with their family in terms of lack of understanding, negative control, unrealistic expectations, guilt or painful experiences. Various participants described difficult childhood experiences, such as parental substance use or mental illness, sexual abuse and violence. Some described their current partner as a support, while others described violence and abuse from their current or former partners. Receiving support from FACT in being a good parent was described as crucial by those who had children, even when they did not currently have custody of the child.

Participants found that team members had complementary roles, which related to different aspects of the participants' lives, leading to an overall experience of being viewed as a whole person. Several participants felt freer and less awkward when relating to a number of people, and some described this as being part of a community. Further, a stable, continuous relationship with one or two members of the FACT team was highly valued by most participants. These were often the case manager, peer support worker or psychologist. By contrast, having to change contact persons was described as starting the process of building trust and sharing information from scratch. One participant put it this way, based on former experiences in the service system:

“*I'm skeptical of new staff. That's what I've been used to. If you're unlucky, like most people are, you get a real bastard who looks down on you and doesn't even bother to talk to you when you call him.”*

The peer support worker was described as someone who was easier to trust, share information with, take advice from, and feel understood by than other professional helpers. Music therapy was described as motivating, allowing for self-expression, a feeling of community, development as a musician, and the possibility to challenge oneself and build social confidence, but it was also felt to be valuable in itself because it was fun. One participant described how she raised her head more in public after having played in a concert with the music therapist, and that several people had come over to talk to her after the concert. Access to a psychiatrist in the team had allowed for better monitoring of medication. Further, access to psychological treatment and assessment had led to more accurate diagnosis. Some participants wanted more diagnostic assessment and did not agree with their current diagnosis. Several stated that they would have preferred more contact with a psychologist or a psychiatrist in FACT. A number of participants mentioned that it took too long to replace the psychologist, music therapist or peer support workers when these had quit, and some felt that nurses and psychiatrists were valued more by management. One participant described his contact with the peer support worker like this:

“*I think things carry more weight when it's someone who's been through it. I realize that other people can understand too, but they're incapable of feeling what I'm going through, you know. When I feel mentally broken and just want to take drugs to feel better. When I'm not ok, you see.”*

A lack of knowledge of substance use among staff had led to substance use problems not being addressed, and to an overly restrictive practice regarding medication, resulting in an experience of not being seen as a whole person. Others described how FACT had helped them by focusing on substance use in a competent way. One participant had experienced negative consequences due to a lack of knowledge about eating disorders among team members.

Collaboration with services outside of FACT had given participants an increased sense of control over their lives and fewer hassles. Collaboration with staff in supported housing was described as having led to adapted routines and fewer conflicts with staff. Furthermore, contact with home-based services had improved medication management and collaboration with welfare services had enabled participants to access services they had wrongfully been denied. This was particularly valuable in periods of poorer mental health when it was difficult to attend meetings, keep track of registration deadlines or order important documents. Several participants wanted better collaboration with housing services, and some participants did not have a resource group of their informal network despite wanting one. Participants varied in their descriptions of collaboration with GPs. Several participants had very little contact with their GP. One participant with diabetes reported that he had not seen his GP for several years, and did not take medication. One participant described collaboration between FACT and his GP in this way:

“*I can see it's a lot easier when you get help from someone who cares, you know. I'm not too keen on doctors, because they brush me off right away. (…) But as soon as other people are involved, they behave differently. Sure, some of them still act like Nazis, but it gets a lot easier.”*

### Being Empowered and Involved

Participants described that FACT had supported citizenship by reinforcing empowerment and involvement. Being empowered and involved in decisions in their contact with FACT had changed participants' attitudes toward themselves and what they expected from services, from feeling that they did not deserve help, to thinking that they were worth a decent life and being part of society. Participants described noticeable changes since coming into contact with FACT, such as increased focus on their wishes and needs, less focus on illness, and less top-down communication. Many participants expressed gratitude toward FACT and felt that they had received a lot more than they would ever have expected, and that they felt lucky, for once in life. One participant said the following:

“*I've always been the kind of person who gives up his place to others. (…) But the doctor said: “You know what, it's actually your turn now.” So I went along with that. It's my turn. And I'm a bit ashamed of it, because it sounds very selfish.”*

Participants described feeling in a vulnerable and disempowered position as patients with severe mental illness. Suspiciousness, being quiet and scared, having trouble expressing themselves, and lack of trust were described as barriers to participation, making it particularly important that the FACT team members actively asked for their opinion, provided information and involved them in decisions. A sense of trust, security and mutual respect were described as necessary for genuine participation. Several participants said that it had been difficult to trust FACT in the beginning due to former experiences of coercion, being treated badly, lack of participation, condescending attitudes and previous unprofessional behavior from health personnel, feeling disempowered, being treated like an object, and treatment being irrelevant and of little help. Some stated that experiences from the substance use environment or other relationships had led to a lack of trust in other people. Many participants said that they had gradually become convinced that FACT was there to help them and not to do them harm. One participant still did not trust FACT because based on her past experiences, if something was too good to be true, it probably was, so she was still waiting for the hidden agenda to become apparent. Trust could be weakened if team members forgot appointments or did not follow up on plans. Some participants felt that team members could not fully understand their situation because of differences between them. One said:

“*Because we're quite apathetic, if we enter a FACT system. We mostly feel that the service system is shit. Because that's how they've treated us and made us feel. Admissions to hospital, coercion, all those things, they make people feel like shit.”*

Another participant said:

“*I eventually had to recognize that they're actually here to help me. They aren't here to hurt me. (…) That reassures me. And then I can trust those people, and that means a lot.”*

Participants found it easier to trust FACT when they perceived the team members to be reliable, respectful, safe, professional, good listeners, helpful, caring, able to disagree in a respectful way, endure their difficult moments, make positive changes and be continuously supportive. The way that FACT worked was described as natural, secure and pleasant. Team members showing who they are as people, engaging in two-way communication, talking about everyday issues, creating an inclusive atmosphere, not being condescending, using emojis, sitting down for a coffee or a cigarette, were all described by the participants as factors that enhanced their relationship. One participant said that it was nice not to feel interrogated when she met with staff. Another participant described the FACT team members in this way:

“*There are people here, they're not robots!”*

Many participants described a high degree of involvement in deciding treatment goals, content, intensity, and individual adjustment. However, some had experienced that important decisions were made by FACT without their involvement, or against their wishes, such as decisions on housing, education, loan applications or job seeking. This had given participants the impression that the team members had lower expectations than they had themselves, which was felt to be unfair and disempowering. Several participants mentioned that former or actual substance use was used against them, making team members trust them less. Several participants wanted more information about what FACT could offer, expected progress, diagnosis and treatment effect. Several were unfamiliar with their treatment plan. One participant said that he wished FACT would avoid using difficult language, as he was often left wondering about things after meetings. Another participant described the importance of being involved:

“*I mean when it comes to going out, being allowed to choose your own path, I think it's very important that people listen to you. And if they don't want to listen, you just die inside.”*

Being involved in decisions about their medication was important to many participants. Some found that they were not listened to because of substance use problems. Others felt that this was better in FACT than before, because team members knew them better. Some participants were content with how medication was handled in FACT, while others wanted a better dialogue with the psychiatrist. Those who felt disempowered concerning medication described considerable frustration, impaired self-esteem, and a negative impact on their lives. One participant went to bed angry and upset every night thinking about the side effects of medications. Another participant had been sad because she had lived with untreated delusions for several years, and was grateful for finally receiving suitable medication. A woman described her experience of disempowerment like this:

“*I've reached an age when I want to be left in peace, to find a way of taking care of myself without medication. But I'm not allowed to, I'm not in a position where they'll listen to me. The system is so huge.”*

Some participants found that while FACT wanted to apply democracy, this was difficult because they were part of an authoritarian system. Being subject to coercion was described as brutal, tough and a desperate feeling. The knowledge that FACT had the authority to introduce coercive treatment frightened participants and challenged their trust. Several participants were uncertain whether they were formally subject to coercion or not. Many agreed to take medication in order to avoid coercive treatment, despite experiencing side effects such as weight gain, fatigue, less energy, lack of mental agility, and feeling like a zombie. Many said that while the psychiatrist listened to them in some cases, they had little impact in important matters. One said:

“*Well, they try to be democratic, like a flat structure. I can feel that. But they're part of a larger system, you know. (…) There's something very authoritarian in that structure.”*

Participants described their contact with FACT as empowering in that team members focused on resources and personal skills, facilitated coping and acknowledged accomplishments. This had increased their self-esteem, independence, and skill in setting boundaries, which had made it easier to participate in society. Psychoeducation had provided new insights and tools for mastering life. Team members pushing and challenging them into trying activities or situations that they would rather avoid was described as empowering, even if it could be frightening and uncomfortable, so long as they knew each other well and could decide themselves when to stop. Examples of this were being encouraged by the music therapist to play in concerts, being invited out for walks in the countryside with team members and other service users, being encouraged to participate in planned activities even on bad days, or engaging in exposure therapy for public transport. Participants described how mastering new situations and reaching their goals led to feelings of happiness and victory, self-confidence, and feeling in control, and enabled them to participate in society in other ways, such as going to concerts and hanging out with friends. Some participants wished that team members would challenge them more, for instance in relation to anger management, health behavior such as smoking, or practicing social skills. One participant described her experiences of being empowered like this:

“*I used to feel like I didn't know anything, ‘I'm no good, everything's been messed up'. But FACT has shown me that I have plenty of good points.”*

Another participant said:

“*Being able to express what you're passionate about, and get feedback. Otherwise you die inside. And I feel like that's happening to me now. I feel like there's a little flower inside me, but it doesn't bloom anymore. Because when it was about to bloom, it was told to stay in its pot.”*

Participants felt empowered when team members expressed faith in their potential and ability to contribute and mean something to others. Many participants wanted to turn their negative experiences into a way of helping others. Many felt privileged because they had received help and managed to improve their lives, and wanted to give something back by helping others and showing that change is possible. Receiving support from FACT in applying for education and jobs, being invited to speak at information meetings about FACT, and playing in concerts with the music therapist were all described as valuable. Team members were described as role models because of the way they worked to help others, and this was particularly true of the peer support workers.

“*I used to feel that other people weren't interested in me, that they seemed a little shut off and maybe closed and private when I met them at work. But after six months I don't feel that anymore. I like it when I don't just feel like a burden or uninteresting to people. Because now I have the feeling that people appreciate me. I actually believe they kind of like me (laughs).”*

### Getting Practical and Accessible Help

Participants described that FACT had supported citizenship by providing practical and accessible help. Meeting outside of the office made services more accessible, particularly in periods when participants isolated themselves due to mental illness. Irregular sleeping hours and homelessness also made it difficult to attend appointments at the office. Team members coming to their home and going for a walk or to a cafe together had reduced anxiety and loneliness, allowed them to express their thoughts, and increased hope and a sense of community and well-being. These appointments with FACT made it easier to manage other things, such as going to the grocery store, freshening up, starting the day right, and establishing daily routines—everyday things that meant having a place to be and feeling part of a community and part of society. However, one participant felt it was an invasion of his private life that team members came to his home. Another participant felt uneasy when team members came to her home because she had not visited their home. Most participants stated that team members respected it if they did not want home visits. One said:

“*I tend to isolate myself, particularly in these COVID times. So it feels really good to go for a walk, talk to someone. That combination really gives me a lot. (…) I can get really afraid of going to the grocery store, but after going for a walk and talking, I go to the shop without any problem.”*

Receiving help with transportation had made life easier, solved practical problems, provided solutions to difficult situations, and created a feeling of security for many participants, particularly in the rural areas. Long distances, bad weather and limited public transportation made other means of transportation necessary. Many participants did not have a driver's license and did not know anyone who did. Getting help with transportation made it possible to get to work, school, meetings with welfare services, medical appointments, or appointments for giving urine samples. The time spent in the car was described as meaningful and valuable, and a nice way to meet team members. One participant said that he preferred talking in the car because he had problems with eye contact. A man in a rural context described the importance of transportation this way:

“*It sounds strange that FACT runs a taxi service. But just imagine how valuable the hours are when we're driving. (…) Whether he helps me drop something at the landfill or whatever. The point is that we get that moment together, when we can work.”*

Meeting out of the office was described as relaxing, comfortable and informal. Several participants mentioned that they could express more aspects of themselves as people in this way. Having team members come with them to meetings with schools, activities or employers had enabled them to attend. A number of participants would have preferred more frequent visits, and that team members had more time. Some expressed concern that team members might be overworked and felt that the teams should have more people, decent working conditions, better offices and more credit and support from their leaders. Meetings with FACT were the only thing that kept some participants going, and it could have a negative impact if the team member seemed stressed or in a rush. Meetings with FACT were described as very important by some participants:

“*Meeting with people is important. Maybe you feel like you have no friends. Then you just kind of crawl into a cave. You feel like people around you are taking advantage of you. Now I have someone to go to.”*

Practical help with what was most important in the participants' everyday life had opened the door to new opportunities, led to hope and a feeling of self-worth, and made it easier to make good life choices. Week plans, reminders, and help in keeping one's home in order were described as useful. Help in dealing with regulations, getting testimonials that led to benefits, filling in forms, getting a phone and managing financial problems had increased participants' sense of security and freedom. Having money to spend on a bottle of wine with one's girlfriend, Christmas presents for the nephews or inviting friends or family for dinner had led to enjoyment, increased self-esteem and more reciprocal relationships. For several participants, it had been necessary to solve the financial worries first in order to focus on other interventions.

“*I told them: ‘You know what, I can't handle this. I can't talk about my childhood when I have so much debt that I have almost no money for food, and every day I'm worried about getting a phone call from the debt collectors'. So they helped me to get a deal with welfare services so that they help me to manage my money matters. And that means a lot. Because when you can put stuff like that behind you, you can think about other things, and get rid of all those things that disturb you. And now I've gradually come back to believing that things can get better.”*

Many participants were pleased that FACT could vary the intensity of care and support according to their needs, because these could vary from week to week. It was reassuring to know that the team was easily accessible and could arrive quickly in a crisis, and that they would help them to maintain an overview when life seemed chaotic. Many said that FACT were the only ones they could contact because they did not have a network or because they found it difficult to ask for help from family or friends. One participant described how team members had saved his life after an overdose in his home. Another had been helped to escape from an episode of domestic violence. Participants appreciated being able to send text messages at night, knowing that team members would respond the next day. Participants were reassured by the fact that treatment continued through better and worse periods, relapses to substance use, inpatient treatment, and times when they isolated themselves at home, did not attend appointments, or were in a bad mood. Participants felt that FACT team members were generous and tolerant, which prevented them from being cut off or lost by the system. One said:

“*They don't mind doing many hours of hard work as long as I'm OK. They work for the person sitting in front of them, no matter how hard it is or how many hours it takes. And they choose what they believe is best for me, even if it means more work for them.”*

Another participant said:

“*To me, this has been a matter of life or death. You have to be honest, and that's the way I feel.”*

Having the time to work through substance use or mental health problems had also made participants feel more secure. Knowing that there was time to finish what had been started led to peace of mind, motivation to engage in treatment, the ability to regain a foothold in the community and the belief that change was possible.

“*I've noticed that it is not like ‘get done with it,' and then they throw you out. They take their time and help me with the time that I need to move on.”*

Many participants said that FACT had helped them with more than they expected, such as hiring a container and helping them to empty their apartment, looking after their cat during inpatient treatment, delivering personal items to people they did not want to meet due to risk of relapse, or providing services to their partner before he officially became a FACT client. This was described as crossing boundaries in a good way, and had been meaningful turning points where participants received the help they needed in order to be capable of making bigger changes. Such unexpected help gave participants a new experience of being worthy of an effort, which increased their motivation and hope.

“*Even if I've messed up and failed, they obviously haven't given up on me. That feels good. I'm used to people giving up on me.”*

## Discussion

The aim of this paper was to explore and describe service user experiences of how receiving services from FACT may support or inhibit citizenship. We will discuss the main findings in relation to the “5 Rs” of the citizenship framework—*rights, roles, relationships, responsibilities, and resources*—and the concept of belonging (Ponce and Rowe, 2018).

### Supporting Citizenship

Being viewed as a whole person was described as supporting citizenship in several ways. Integration of different services in one team, and the opportunity to address several aspects of one's life at the same time, had led to better access to *resources*, access to new *roles*, and improved *relationships*. The importance of multiple competences within the team corresponds to the principle of effectiveness of interventions in the EUCOM principles for community-based mental health care (Pieters et al., 2017; Keet et al., 2019). While integration within the team seemed to work well, there seemed to be room for improvement in collaboration with other services and actors in the community outside of the team. This finding is in line with research on personal, social and clinical recovery in severe mental illness, where social recovery seems to stay behind, even when personal and clinical recovery is improved (Castelein et al., 2021). It also coincides with the mixed results from studies of improvement in social functioning among service users in FACT (Drukker et al., 2013; Svensson et al., 2018; Kortrijk et al., 2019a). However, from a relational recovery perspective, personal, clinical and social recovery are closely interrelated and interdependent processes (Price-Robertson et al., 2017). This is supported by participants' descriptions of how labeling and exclusion had affected their opportunities for recovery and citizenship. Addressing meaning in life, empowerment, and social factors in addition to symptom reduction and improved functioning, may lead to better outcome for people with severe mental health problems (Vogel et al., 2020). The engagement of people and communities and mingling health and social services with other community actors such as schools, industry, non-government organizations or other community groups, have been mentioned as priority areas for service integration (Goodwin, 2016).

Being considered as a whole person also involved support in accessing various enjoyable activities in the community. The value of fun and pleasurable experiences in this study is consistent with studies of community approaches other than FACT (Davidson et al., 2006). The particular aspect of walking in the countryside may have a cultural component and increase the sense of belonging in society, as outdoor life and nature experiences are often central in Norwegian everyday life. It may also have to do with the ongoing SARS Cov2/Covid19-pandemic, and the fact that many other activities were canceled. These findings suggest that wilderness therapy, developed for at-risk youth (Fernee et al., 2015), may also be useful for people who receive services from FACT. Diverse and meaningful activities provided *a sense of belonging*, but also strengthened participants' ability to face societal barriers to citizenship, such as stigma and discrimination. The importance of meaningful work and other activities in order to access new *roles, responsibilities* and *a sense of belonging* corresponds with literature on the relationship between occupational meaningfulness, meaningful activities, citizenship, and recovery for people with complex needs (Nordaunet and Sælør, 2018; Nesse et al., 2021).

Team members who adopted a strength-based approach, pushing the participants into exploring new situations, explicitly believing in their potential, and supporting efforts to pursue life goals such as education, work or housing, had improved access to new life *roles* and *resources*, and increased participants' ability to take *responsibility*. Opportunities to grow as a person and to mean something to others were described as supporting citizenship and as meaningful in themselves. Experiences of participants in this study coincide with the concept of “mattering,” or the importance of feeling valued and adding value (Prilleltensky, 2020). Research on the attitudes of service providers has shown that they may express concerns that clients are not well enough to assume responsibilities as equal citizens (Ponce et al., 2016; Brekke et al., 2018b). However, the citizenship framework stresses that issues such as mental health or substance use problems, housing, poverty or legal concerns should not stand in the way of achieving citizenship, and that citizenship is for everyone (Ponce and Rowe, 2018). This resonates with the United Nations International Guidelines on Human Rights and Drug Policy, which stress that human rights apply to everyone, including people with substance use problems who continue to use substances (United Nations, 2019).

Receiving practical and accessible help from the FACT team was described as improving participants' financial situation and access to *resources* such as dental and health care services. Help in solving financial issues had led to more reciprocal *relationships*, feelings of mattering by being able to help others financially, escaping poverty and gaining increased capacity to address other life areas. This points to the importance of integrating social work in a multi-disciplinary team, and is in line with literature that suggests poverty as an important mechanism in mental health problems (Read, 2010; Tew et al., 2012). The value of accessible help supports a pragmatic and person-centered approach to service design, adapting services according to what actually works for service users. Also, this finding supports community-based mental health and substance use services as a means to address the issue of unfair access to mental health (Pieters et al., 2017).

### Stigma and Coercion

Being empowered and involved in decisions related to their lives had supported the participants' experiences of citizenship in different ways. Participants described serious barriers to participation and involvement when getting in touch with FACT, which were related to past experiences of violence, abuse, discrimination, coercion, objectification and disempowerment within and outside of the treatment system. Being related to in ways that made it possible to trust team members along with invitations to be involved in decision-making had made it easier to exercise one's *right* to participate. This is in line with research that has suggested that people with severe mental illness and complex needs need to be able to trust practitioners and services in order to gain genuine access to health and social services (Edland-Gryt and Skatvedt, 2013; Brekke et al., 2018a). Several participants described the phenomenon of “dying inside” in relation to experiences of not being allowed to express oneself. One way of interpreting this is that participation and involvement are existential needs. This implies that while participation and service user involvement may be thought of as a bureaucratic issue by service providers, they may be fundamentally important in the lives of service users. The metaphor of a flower that has stopped blooming that was expressed by one participant resonates with the concept of “flourishing” in positive psychology (Diener et al., 2009), and with perspectives that argue for understanding flourishing and well-being in relation to context and social justice (Di Martino et al., 2017).

Participants described experiences of not being recognized as citizens by colleagues, neighbors or other citizens in their community. This coincides with the concept of “micro-aggressions” (Gonzales et al., 2015). Experiences of being left out and labeled because of mental health or substance use problems are examples of stigmatization (Stuart et al., 2012). Expressions of not deserving a good life, or being worthy of citizenship, may be seen as examples of self-stigma among participants in this study (Watson et al., 2007). Similarly, results in the current study support the importance of “micro-affirmations” (Topor et al., 2018), or small signs of acknowledgment, like when the peer support worker sat down for a coffee and a cigarette, talking about regular things, which was described as “making you feel like a citizen.” Actions to reduce stigma and self-stigma are important elements of recovery-oriented practices (Bejerholm and Roe, 2018). The Scandinavian countries are often considered egalitarian cultures with a high level of social trust, low socioeconomic differences and a low level of individual blame for poverty, which has been associated with a lower level of feelings of inferiority, or “status anxiety” among citizens (Wilkinson and Picket, 2010). This does not seem to be reflected in the accounts of participants in this study, which may suggest that the positive effects of an egalitarian society do not necessarily extend to everyone (Steckermeier and Delhey, 2019). The expressions of self-stigma among participants in this study may be understood in light of the cultural and historical views of substance use problems as self-inflicted, and people with substance use problems and complex needs as “unworthy needy,” which are notions that have been prevalent in Norwegian society (Johansen et al., 2018).

Experiences of coercion and the potential for coercion had inhibited citizenship for some participants in this study. This supports the notion that compulsory treatment is contrary to the recovery goals of living a meaningful life as a recognized citizen in society (Slade et al., 2014). Coercion and the potential for coercion depend on the service and legal systems surrounding FACT, and seemed difficult to address for FACT team members. This illustrates the importance of structural issues in promoting citizenship (Rowe and Davidson, 2016).

In summary, FACT seems to enable citizenship by supporting the person's ability and efforts, by providing holistic and integrated services, enhancing empowerment and participation, and offering practical outreach help adapted to the person's current life situation. There are some examples of involving the community, such as collaboration with employers, professionals outside of FACT and family members. However, the FACT model seems to hold the potential of even more engagement with local communities to improve participation for service users in FACT and other persons with life struggles such as mental illness and complex needs. The results in this study support the need for relational and structural approaches to citizenship (Vandekinderen et al., 2012).

### Strengths and Limitations

A participatory research design does not necessarily improve research quality in a traditional sense (Malterud and Elvbakken, 2019). However, traditional understandings of quality have been contested, stressing the value of involving different sources of knowledge (Koksma and Kremer, 2019). Power imbalance may also raise questions about the genuineness of participation (Sangill et al., 2019). In the current study, advice from the peer group in the planning of the study led to changes in the recruitment strategy, the interview setting, and the wording of the interview guide, which we believe made participation easier for some participants.

The participatory design also provided for the understanding and representation of the sub-cultural context of living with mental illness and substance use problems. Previous studies have reported that interviewers with lived experience can make it easier to establish trust during interviews (Veseth et al., 2019), elicit richer descriptions, hence increasing the quality of the data (Barber et al., 2011), and access information that would otherwise not have been shared. This resonates with experiences from the current study, as several participants mentioned that they appreciated that one of the researchers had service user experience. Being two interviewers with different backgrounds also helped us complement each other's perspectives during the interviews, and to reflect on the interview situation and content after each interview. For instance, the 3rd author asked follow-up questions based on lived experience of the phenomena and knowledge of the sub-cultural context that elicited new information as well as elaborations of previous descriptions, allowing for a deeper understanding of participants' experiences.

Service user involvement and co-production in the analysis process has been described to enhance complexity (Mjøsund et al., 2017), identify new themes, increase relevance and communication of the results (Barber et al., 2011), and increase credibility of the results (Pettersen et al., 2019; Veseth et al., 2019). In the current study, involvement of the peer group and co-researcher with lived experience led to a more nuanced and deeper understanding of the results, as well as decisions during analysis of greater relevance to the field. The power imbalance was addressed by making sure that all involved received financial compensation, creating an equal setting for the meetings, avoiding difficult language, and clearly defining roles and decision-making power from the beginning. The first author's decisions were strongly influenced by advice from the peer group, but also by issues of academic quality and pragmatic feasibility. This made power structures more transparent, but also implied a lower level of participation than that found in other collaborative studies, such as user-led research (Beresford and Carr, 2012).

Four participants chose to have a team member present during the interview, which may have influenced the results. One participant had only been in contact with the team for the relatively short period of four months, which may also have influenced the results. Further, the use of a digital platform and telephone to conduct some interviews may have affected the results. The fact that data collection took place during the COVID-19 pandemic might have affected the opportunities for community life and citizenship, as well as the interventions from the FACT teams. One strength of this study is the variation among participants, and the fact that they lived in five different geographical contexts. While the contexts are varied, the five teams are not representative for all FACT teams in Norway. All authors have Western European background, which may have influenced the methods and analyses. The methods do not allow for an immediate generalization of the results, nor for comparison between the different geographical and cultural contexts. However, we would argue that the methods allow for a deeper understanding of how FACT may be experienced by service users to support or inhibit citizenship, and that this understanding may be valid in other contexts and for other people.

## Conclusions

The evidence from the experiences of service users of FACT with severe and complex mental health problems in this article suggests that many of them have experienced a lack of citizenship, and that FACT may play a crucial role in promoting citizenship. Coercion, lack of involvement, and authoritarian aspects of the system surrounding the FACT team may inhibit citizenship. While the efforts of the persons themselves and the FACT team members are necessary in the process of regaining citizenship, recognition from the community also appears as a necessary factor. Results from this study suggest that FACT teams support service users' efforts and capacity in pursuing citizenship, and that FACT teams have a potential to increase efforts to reduce barriers to citizenship in community and society. This study generally supports the need for relational, inclusive and structural approaches in order to support citizenship for people with severe mental illness and complex needs. There is a need for research that investigates how FACT and other services may support citizenship in a broader sense, in collaboration with service users, policy makers, and local communities.

## Data Availability Statement

The raw data supporting the conclusions of this article will be made available by the authors, without undue reservation.

## Ethics Statement

Ethical review and approval was not required for the study on human participants in accordance with the local legislation and institutional requirements. The patients/participants provided their written informed consent to participate in this study.

## Author Contributions

EB has contributed in the planning of the study, recruitment of participants, data collection, analysis, and writing of the article. HC has contributed in the planning of the study, analysis, and writing of the article. MB has contributed in the planning of the study, data collection, analysis, and writing of the article. AL has contributed in the planning of the study, with input to recruitment and analysis, and in writing the article. RK has contributed in the planning of the study, with input in the analysis process, and in writing the article. CM has contributed in planning the study, and in writing the article. ASL has contributed in planning the study, in recruitment, in the analysis, and in writing of the article. All authors contributed to the article and approved the submitted version.

## Funding

This study was funded by the Research Council of Norway, grant number 288722.

## Conflict of Interest

The authors declare that the research was conducted in the absence of any commercial or financial relationships that could be construed as a potential conflict of interest.

## Publisher's Note

All claims expressed in this article are solely those of the authors and do not necessarily represent those of their affiliated organizations, or those of the publisher, the editors and the reviewers. Any product that may be evaluated in this article, or claim that may be made by its manufacturer, is not guaranteed or endorsed by the publisher.
